# Complex Microbiome in Brain Abscess Revealed by Whole-Genome Culture-Independent and Culture-Based Sequencing

**DOI:** 10.3390/jcm8030351

**Published:** 2019-03-12

**Authors:** Jyun-Hong Lin, Zong-Yen Wu, Liang Gong, Chee-Hong Wong, Wen-Cheng Chao, Chun-Ming Yen, Ching-Ping Wang, Chia-Lin Wei, Yao-Ting Huang, Po-Yu Liu

**Affiliations:** 1 Department of Computer Science and Information Engineering, National Chung Cheng University, Chia-Yi 62102, Taiwan; twolinin@gmail.com; 2 Department of Veterinary Medicine, National Chung Hsing University, Taichung 40227, Taiwan; zongyen@gmail.com; 3 Genome Technologies, The Jackson Laboratory for Genomic Medicine, Farmington, CT 06032, USA; Liang.Gong@jax.org (L.G.); CheeHong.Wong@jax.org (C.-H.W.); 4 Department of Medical Research, Taichung Veterans General Hospital, Taichung 40705, Taiwan; cwc081@hotmail.com; 5 Program in Translational Medicine, National Chung Hsing University, Taichung 40227, Taiwan; chunmingyen@gmail.com; 6 Department of Neurosurgery, Neurological Institute, Taichung Veterans General Hospital, Taichung 40705, Taiwan; 7 Department of Otolaryngology-Head and Neck Surgery, Taichung Veterans General Hospital, Taichung 40705, Taiwan; entcpw@gmail.com; 8 Division of Infectious Diseases, Department of Internal Medicine, Taichung Veterans General Hospital, Taichung 40705, Taiwan

**Keywords:** metagenomics, whole genome sequencing, genomics, brain abscess, *Streptococcus constellatus*, *Prevotella*

## Abstract

Brain abscess is a severe infectious disease with high mortality and mobility. Although culture-based techniques have been widely used for the investigation of microbial composition of brain abscess, these approaches are inherent biased. Recent studies using 16S ribosomal sequencing approaches revealed high complexity of the bacterial community involved in brain abscess but fail to detect fungal and viral composition. In the study, both culture-independent nanopore metagenomic sequencing and culture-based whole-genome sequencing using both the Illumina and the Nanopore platforms were conducted to investigate the microbial composition and genomic characterization in brain abscess. Culture-independent metagenomic sequencing revealed not only a larger taxonomic diversity of bacteria but also the presence of fungi and virus communities. The culture-based whole-genome sequencing identified a novel species in *Prevotella* and reconstructs a *Streptococcus constellatus* with a high GC-skew genome. Antibiotic-resistance genes *CfxA* and *ErmF* associated with resistance to penicillin and clindamycin were also identified in culture-based and culture-free sequencing. This study implies current understanding of brain abscess need to consider the broader diversity of microorganisms.

## 1. Introduction

Brain abscess is one of the most life-threatening infectious diseases, commonly resulting from contiguous spread from an adjacent infected focus [1]. Despite diagnostic and therapeutic advances, the mortality remains high—from 15 to 85% [1]. The reported causative organisms vary depending on the clinical circumstances. Brain abscess may be caused by bacteria, fungi and parasites [1]. In most cases, the detection of causative organisms is made by culture of drainage abscess. However, culture-negative brain abscess is not uncommon, occurring in between 9% and 63% of patients in difference series [2,3,4,5]. Hence, the microbial spectrum involved in brain abscess is incompletely characterized.

The applications of advanced sequencing technology enable culture-independent approaches for the detection of difficult-to-culture or unculturable taxa. There are several studies that have been conducted using 16S rRNA gene amplicon sequencing to characterize bacterial community in brain abscess [6,7,8,9]. While these studies have detected microbial signatures of brain abscess, they are based on the amplification of a specific gene of bacteria, leaving other microbial community (i.e., fungi and virus) largely unexplored [10]. For instance, fungi have also been identified in brain abscesses, showing that investigation of microbiome needs to extend beyond the typical bacterial 16S rRNA gene sequencing.

The development of culture-independent whole-genome metagenomic sequencing offers an unbiased approach for investigating the entire microbial community as well as the genetic factors in the genomes [11]. The whole-genome metagenomic approach to clinical specimens randomly samples DNA en masse. In contrast, targeted approaches using specific amplification are biased to the detection of a subpopulation and a small subset of genetic elements [12]. 

The present study applied both culture-independent and culture-based sequencing for comprehensively characterizing brain abscess microbial communities. Complete genomes of cultured isolates were reconstructed and analyzed using a combination of Illumina and MinION Nanopore sequencing. Culture-independent metagenomes were sequenced by Nanopore platform, disclosing a larger diversity of microorganisms including bacteria, fungi and virus.

## 2. Materials and Methods

### 2.1. The Case

**Figure 1:**
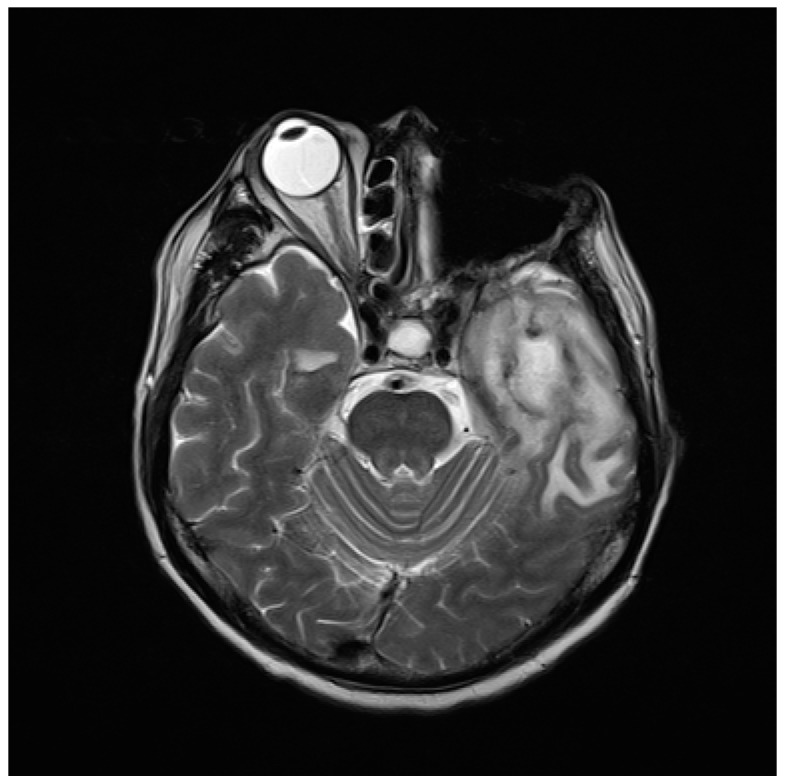
Magnetic resonance image (MRI) of brain showing heterogeneous enhancement with focal rim enhancing cystic lesions in left anterior temporal lobe.

A 60-year-old man presented with fever and headache. He had a history of adenoid cystic carcinoma of left maxillary sinus and received left total maxillectomy, orbital exenteration, external ethmoidectomy and sphenoidectomy. The MRI revealed heterogeneous enhancement with focal rim enhancing cystic lesion surrounding with perifocal edema, indicating brain abscess (Figure 1). This brain abscess specimen was collected by stereotaxic aspiration. The patient consented the aspirates sent for conventional microbiology survey and metagenomic analyses. This research was approved by the Institutional Review Board of Taichung Veterans General Hospital (CE16111B).

### 2.2. Conventional Bacterial Strain Identification and Susceptibility Test

Aspirate from the abscess was subjected to culture. The specimens was inoculated onto Trypticase-soy agar supplemented with 5% horse blood (bioMérieux, Marcy l’Etoile, France), chocolate agar plate (bioMérieux, Marcy l’Etoile, France) and eosin-methylene blue agar plate (Becton Dickinson, Sparks, MD, USA). ±1 mL of the sample was inoculated in thioglycolate broth (bioMérieux, Marcy l’Etoile, France) for enrichment. Agar plates and broth were incubated aerobically at 37 °C. For anaerobic culture, CDC anaerobe agar plate with 5% Sheep Blood (Becton Dickinson, Sparks, MD, USA) were used. The plate was incubated fat 37 °C in anaerobic condition. Preliminary identification of isolates were performed using the Vitek 2 system (bioMérieux, Marcy l’Etoile, France) and the Sanger sequencing of 16S rRNA amplified by PCR using universal primers 27F(5′-AGAGTTTGATCCTGGCTCAG-3′) and 1492R(5′-TACGGYTACCTTGTTACGACTT3′). 16S rRNA gene-based identification reported *Streptococcus* sp. (Designated TCV107) and *Prevotella* sp. (Designated TCVGH). The in vitro susceptibility of the isolates were determined by the Vitek 2 system (bioMérieux, Marcy l’Etoile, France).

### 2.3. DNA Extraction

Genomic DNA of *Streptococcus constellatus* TCV107 and *Prevotella* sp. TCVGH was prepared from overnight liquid cultures grown in MAS (Medium for Acinetobacter Supplemented) broth at 30 °C with shaking to an O.D.600 of approximately 1.5. Cells were pelleted and lysed in the presence of Lysozyme from chicken egg white (Sigma, St. Louis, MO, USA). Genomic DNA was purified by phenol-chloroform (Sigma) phase extraction. Extracted DNA was resolved in 100 μL TE buffer (10 mM Tris, 1 mM EDTA [pH 8.0]) supplemented with 10 μg/mL RNase (Sigma).

### 2.4. Illumina Library Preparation and Sequencing of Isolates from Brain Abscess

DNA (30–100 ng) was sonicated to a 100–800 bp size range using a Covaris E210 sonicator (Covaris, Woburn, MA, USA). Fragments were end-repaired, 3′-adenylated and Illumina adapters were then added using the NEBNext Sample Reagent Set (New England Biolabs, Ipswich, MA, USA). Ligation products were purified using Ampure XP (Beckmann Coulter Genomics, Danvers, MA, USA) and DNA fragments (>200 bp) were PCR amplified using Illumina adapter-specific primers and Platinum Pfx DNA polymerase (Invitrogen, Carlsbad, CA, USA). Amplified library fragments of 650–750 bp were size selected on a 3% agarose gel. Libraries were quantified by qPCR using the KAPA Library Quantification Kit for Illumina Libraries (KapaBiosystems, Wilmington, MA, USA) and library profiles were assessed using a DNA High Sensitivity LabChip kit on an Agilent Bioanalyzer (Agilent Technologies, Santa Clara, CA, USA). Libraries were sequenced on an Illumina MiSeq instrument (San Diego, CA, USA) using 300 base-length read chemistry in a paired-end mode.

### 2.5. Nanopore Library Preparation and Sequencing of Isolates from Brain Abscess

Library preparation was performed using the 1D Genomic DNA sequencing kit SQK-LSK108 (Oxford Nanopore Technologies) with the omission of DNA shearing and DNA repair steps to prevent further DNA fragmentation. Library preparation was initiated at the DNA end-prep step. All cleanup steps were performed using AMPure XP beads (Beckman Coulter). The final 80 µL prepared library was proceeded to sequencing on the MinION Mk1b device using a FLO-MIN-106 R9.4 flow cell (Oxford Nanopore Technologies, Oxford, UK) using the MinKNOW software for the full 48 h run time with no alterations to any voltage scripts.

### 2.6. Metagenomic Sequencing

Aspirated abscess was diluted in 1 mL 0.9% sodium chloride. The sample was sedimented by centrifugation at 500 g for 5 min at 4 °C. The supernatant was centrifuged again at 800 g for 5 min at 4 °C for the separation of the human cells. DNA was extracted with standard silica mini-columns (Qiagen Genomic-tip 20/G) following the manufacturer’s instruction. DNA purity and concentration were determined using Nanodrop (NanoDrop 2000, Thermo Fisher Scientific, Waltham, MA, USA). Approximately 400–500 ng DNA was taken to construct a DNA library for nanopore sequencing using a Rapid Sequencing Kit (SQK-RAD003 from Oxford Nanopore Technologies, Oxford, UK) as described by the manufacturer and then loaded onto a MinION Mk1b device using a FLO-MIN-106 R9.4 flow cell (Oxford Nanopore Technologies, Oxford, UK) following the standard 48-h run scripts.

### 2.7. Genome Assembly and Gene Annotation

The TCV107 and TCVGH isolates were sequenced by both Nanopore and Illumina platform. The sequences were assembled using Canu v1.5 [13] and SPAdes v3.11.1 [14] software. The assembled genome were further polished using Racon v1.3.1 [15] followed by Nanopolish v0.9.0 [16]. Finally, the polished genome was circularized using Circlator v1.5.5 [17]. Species identification were conducted via MIGA and BLAST scan of NCBI microbiome database, indicating presence of *Streptococcus constellatus* TCV107 and *Prevotella* sp. TCVGH. Gene annotation was performed via National Center for Biotechnology Information (NCBI) Prokaryotic Genomes Automatic Annotation Pipeline (PGAAP). The TCVGH and TCV107 genomes as well as annotations have been deposited in NCBI with accession numbers QFFX00000000 and CP029207. GC skew was calculated using a 10kb window sliding along the entire genome for calculating (C − G)/(C + G) ratio. In order to determine the loci of *ori* and *ter* in TCV107, we extracted *ori* and *ter* sequences from *Streptococcus pyogene* M3 genome (ori: ~1,650 kb-230 kb, ter: ~920-~1100 kb) and mapped them onto the *Streptococcus constellatus* TCV107 genome by BLAST.

### 2.8. Taxonomic Classification

The de novo assembled genomes indicated novel species (e.g., *Prevotella* sp. TCVGH) not presented in existing NCBI microbiome database. As the sequences are a mixture of human and unknown microbiome, we construct a more comprehensive database by integrating the human genome, NCBI microbiome database and de novo assembled genomes from culture-based sequencing. Minimap2 was used to align culture-free ONT reads against the new integrated database. Because short reads lack specificity during classification, reads with a length less than 500 bp were discarded. In addition, reads with insufficient alignment coverage (<70% of the original read length are also filtered. The top hits of the remaining read alignments were extracted to plot the taxonomic classification. Both cutoffs were determined by statistics of read length and coverage (Supplementary Figure S1).

### 2.9. Identification of Antimicrobial-Resistant Genes and Virulence Factors

The Comprehensive Antibiotic Resistance Database (CARD) [18] and the virulence factor database [19] were used for annotation of virulence and antimicrobial factors. The method was further optimized separately for culture-based and culture-free sequencing. In the culture-free sequencing, the resistome of TCV107 and TCVGH were annotated by using the Resistance Gene Identifier (RGI) from CARD. RGI prediction of resistome is based on homology and SNP models. In homolog models, BLAST is used to detect functional homologs of antimicrobial resistance genes. In contrast, SNP models identify candidate genes which acquire mutations conferring antimicrobial resistance genes based on curated SNP matrices. The virulence factors were predicted through BLAST alignments against the VFDB database using a 95% sequence identity threshold. In the antibiotic-resistance genes annotation of culture-free sequencing, ONT reads were mapped onto antibiotic-resistance genes in CARD by BLASTX. Due to the high error rate of ONT reads, we observed a lot of false-positive alignments. In order to achieve higher specificity than sensitivity, the threshold of alignment identity is set to 100% and e-value less than 1.

## 3. Results

**Figure 2:**
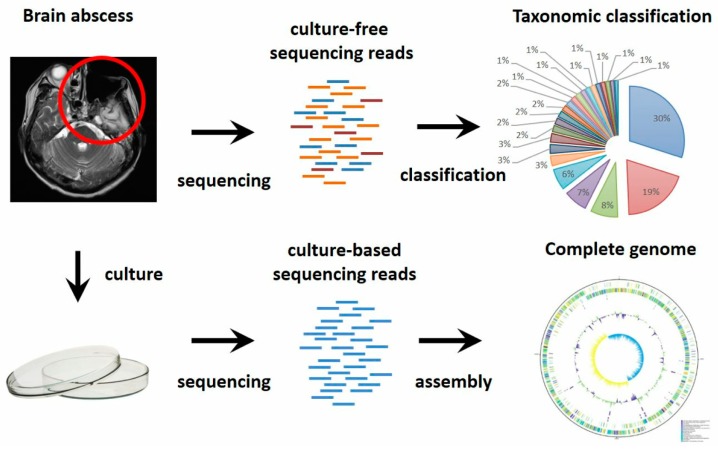
Experimental workflow of culture-based and culture-free sequencing. Brain abscess was first confirmed by MRI. The abscess sample was cultivated and followed by sequencing and assembly. The sample was also directly sequenced in order to disclose the taxonomic composition of entire community including uncultivated bacteria.

The cultured isolates were sequenced using Nanopore and Illumina platforms and culture-free metagenomic sequencing of brain abscess sample was performed using Nanopore platform (Figure 2, Supplementary Tables S1 and S2). Culture-based sequencing reconstructed entire bacterial genomes from isolates, which provided genomic features and insight into taxonomy, virulence and antibiotic-resistant determinants. On the other hand, culture-free sequencing is essential to unbiasedly reveal complex microbial communities, in particular with regard to uncultivated organisms.

### 3.1. Novel Bacterial Genome Revealed by Culture-Based Sequencing

**Figure 3:**
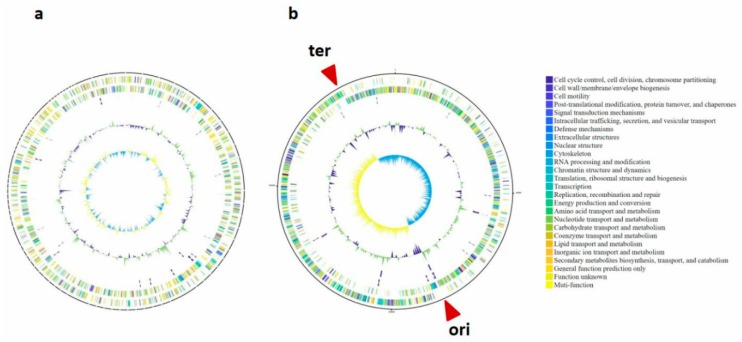
Circular genome map of *Prevotella* sp. TCVGH (**a**) and *Streptococcus constellatus* TCV107 (**b**). Circular genome maps. From outer to inner circles: genes on forward strand, genes on reverse strand, rRNA, GC content and GC skew. (**a**) Circular genome map of *Prevotella* sp. TCVGH; (**b**) Circular genome map of *Streptococcus constellatus* TCV107. The innermost circle shows strong and opposite GC skews in the leading and lagging strands, partitioning the genome into two halves separated by replication origin (*ori*) and terminus (*ter*) sequences. The two outer circles exhibit strong gene orientation bias, whereas half genes are dominantly on the forward strand and the other half are on the reverse strand, partitioned by *ter* and *ori*.

Through hybrid sequencing and *de novo* assembly of cultured isolates, we obtained two whole genomes: a 3.06Mb *Prevotella* sp. TCVGH and a 1.96Mb *Streptococcus constellatus* TCV107 (Figure 3 and Table 1). The TCVGH genome is most similar to *Prevotella enoeca* genome, yet only 70.29% average nucleotide identity (ANI) value (Figure 3a, Supplementary Figure S2). Consequently, it was classified as a novel *Prevotella* subspecies (Prevotella sp.) under the *Prevotellacea* family (*p* = 0.0018) and likely belonging to the *Prevotella* genus (*p* = 0.31). The complete genome of TCV107 was unambiguously classified as *Streptococcus constellatus* (with 97.03% ANI). The TCV107 genome exhibits prominent GC-skews (Figure 3b). The two skew breakpoints contain the replication origin (*ori*) and terminus (*ter*) sequences, which divides the genome into two halves possibly corresponding to replichores. The replication leading and lagging strands are dominant with nucleotide G and with C, respectively. In addition, protein-coding genes are largely presented on the replication leading strand and usually absent on the lagging strand. Functional annotation of the TCVGH and TCV107 genomes revealed 2569 and 2008 protein-coding genes, respectively. Gene functions were grouped into 25 categories according to Clusters of Orthologous Groups (COGs) (Supplementary Tables S3 and S4). Replication, recombination and repair genes are the major category in TCVGH genome, while genes involving carbohydrate transport and metabolism are the dominant class in TCV107 genome.

### 3.2. Complex Communities of Bacteria, Fungi and Virus Revealed by Culture-Independent Metagenomic Sequencing

**Figure 4:**
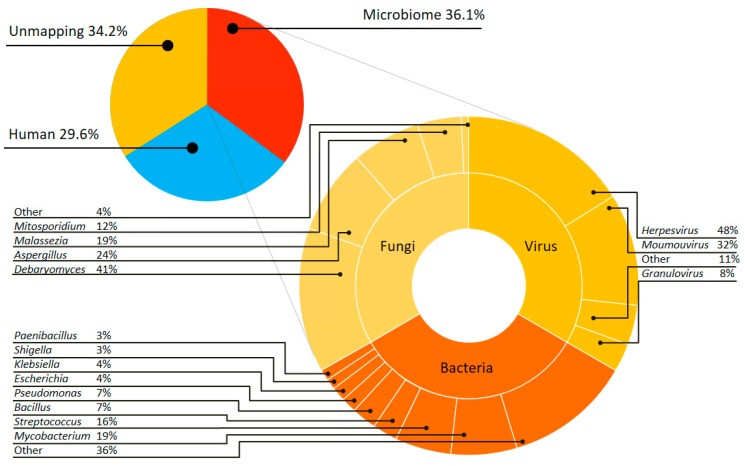
Microbial composition of brain abscesses. Taxonomic classification of culture-free sequencing. In total, microbiome occupies 36.1% while human contamination and unmapping reads takes 29.6% and 34.2%, respectively. The microbiome reads are further classified into bacteria (97.5%), fungi (1.5%) and virus (1%) communities. The species within each community are shown in the outer circle.

Culture-free metagenomic sequencing of the brain abscess sample disclosed a complex microbial community. Using strict read-mapping and taxonomic-classification criteria (see Method), we recovered ~36% of microbiome reads from the metagenomic sequencing, whereas human sequences occupied 29.6% and the remaining reads were unmapped to neither human genome nor any genome in NCBI microbiome database. Taxonomic classification of microbiome reads revealed not only a larger bacterial community but also the communities of fungi and virus (Figure 4).

The bacterial community is composed of more than ten genera (Supplementary Table S5). Although the two cultured isolates (i.e., *Streptococcus constellatus* TCV107 and *Prevotella* sp. TCVGH) were successfully re-discovered, the most-frequent bacteria is *Mycobacterium* (~19%), followed by *Streptococcus* (~16%) and the assembled *Prevotella* genome only takes ~0.24% in the community. The fungi community is dominant by *Debaryomyces* (41%), followed by *Aspergillus* (24%), *Malassezia* (19%) and *Mitosporidiym* (12%) (Supplementary Table S6). The virus community consists of *Herpesvirus* (48%), *Moumouvirus* (32%) and *Granulovirus* (8%) (Supplementary Table S7). To our best knowledge, this is the first report of the existence of both fungi and virus communities in brain abscess, implying different clinical treatments may be required and the need for culture-free sequencing.

### 3.3. Antibiotic Resistance Genes Revealed by Culture-Based and Culture-Independent Metagenomic Sequencing

*Streptococcus constellatus* TCV107 was susceptible to all tested antibiotics, including penicillin, vancomycin, clindamycin and erythromycin, whereas *Prevotella* sp. TCVGH was susceptible to metronidazole, cefoxitin and chloromycin but resistant to penicillin and clindamycin (Supplementary Tables S8 and S9). By using antibiotic-resistant database (CARD) and virulence factor database (VFDB), we identified four antibiotic-resistant genes (*ErmF*, *tetQ*, *CfxA3*, *CfxA2*) in TCVGH genome and two virulence factors (*hasC* and *psaA*) in TCV107 genome (Table 2, Supplementary Table S10). The antibiotic-resistance genes are all encoded on the TCVGH chromosome, of them *CfxA2*/*CfxA3* and *ErmF* are known penicillin- and chloromycin-resistant determinants, respectively. Finally, we assess the sensitivity of antibiotic-resistance genes detection in culture-free sequencing of the same abscess sample, which is mixed with human cells. While missing *CfxA* and *ErmF*, *tetQ* was successfully rediscovered in the metagenomic sample (Supplementary Figure S1).

## 4. Discussion

The study reveals a highly diverse microbial population in brain abscess. Our findings are complementary to those of previous studies for bacteria [6,20]. The dominant reads of Bacteria domain were related to *Streptococcus*, *Pseudomonas* and *Escherichia* (Supplementary Table S6). *Streptococcus* is a common pathogen in head and neck infections and has been associated with brain abscess caused by contiguous spread from ear or paranasal sinus [21]. *Pseudomonas* and *Escherichia* have been reported in brain abscess related to surgical procedure and paranasal sinus infection [22]. The bacterial spectrum is consistent with the host factors and reflects the polymicrobial nature of brain abscess resulting from parameningeal foci of the head and neck and previous surgical procedures.

Among the Fungi domain, the three most dominant genus were *Debaryomyces, Aspergillus* and *Malassezia*. The association of *Debaryomyces* with central nervous system infection has been reported [23]. *Aspergillus* and *Malassezia* are difficult-to-culture commensal of skin and known pathogens of brain abscess [24,25]. In this sense, the association of *Aspergillus* and *Malassezia* with brain abscess could be related to damage of the natural protective barriers [24]. *Herpesvirus* is the most dominant virus type in the study and one of the most common viral causes of encephalitis [26].

Depending on the employed method, the detected microbial communities were significantly different. Although culture-based techniques have been widely used for the investigation of microbial composition of brain abscess, these approaches are inherently biased by the media utilized for growth, result in limited data available on the spectrum of causative organisms. 16S rDNA-based targeted sequencing revealed unculturable bacteria but cannot identify fungus and virus. Shotgun metagenomics provides opportunities explore a microbial community with a wide context.

In *Streptococcus constellatus* TCV107 the two regions of GC skew divide the genome into two halves and the two breakpoints contain the replication origin (*ori*) and terminus (*ter*) sequences, which possibly corresponding to two replichores. This implies the GC skew is likely correlated with DNA replication [27]. Furthermore, protein-coding genes are also partitioned into two halves by *ori* and *ter*, whereas one half is largely presented on the forward strand, while the other half are mostly on the reverse strand. Strong nucleotide composition bias and gene-orientation bias were previously found in intracellular parasites [28].

Of the four antibiotic-resistance genes found in TCVGH genome (i.e., *CfxA2*, *CfxA3*, *ErmF*, *tetQ*), *CfxA2* and *CfxA3*, are likely the main factors leading to penicillin resistance [29]. In addition, *ErmF* is a known chloromycin-resistant determinant, which can alter the binding site of clindamycin via encoding an erythromycin resistance methylase [30]. *tetQ* often accompanies with *ErmF* and associated with reduced susceptibility to tetracycline in other *Prevotella* species.

The large fraction of unmapped reads were also reported in References [31,32,33] and partly due to the incompleteness of existing database biased against uncultivated microorganisms [34]. Though not emphasized in the paper, we ever assembled the metagenomic reads in the hope of reconstructing uncultivated microorganisms. But due to insufficient DNA from clinical sample, only five small contigs (two human and three plasmids) were assembled. Hence, taxonomic classification of this study was still mainly based on existing microbiome database, which may miss uncultivated microorganisms. Another limitation is the possible contamination. Contamination from reagents and device do impact sequence-based microbiome analyses [35], in particular for sample types that have low microbial biomass [36]. Some experts suggest concurrent sequencing of negative control samples [35] and strict laboratory condition to reduce the impact of contaminants [37].

## 5. Conclusions

In summary, we showed that metagenomic sequencing was concordant with the conventional culture-based method. Moreover, the unbiased nature of metagenomic DNA sequencing allowed an expanded scope of pathogen detection including bacteria, fungal species and viruses, while concurrently providing drug resistance information. These data suggest a practical diagnostic decision tree whereby samples negative by routine culture are then advanced to both metagenomic sequencing. This approach will not only complement the current diagnostic paradigm but also allow for a more comprehensive characterization of the etiology of brain abscess.

## Figures and Tables

**Table 1:** Genomic data of *Prevotella* sp. TCVGH and *Streptococcus constellatus* TCV107.

Species	Genome Size (bp)	GC Content (%)	Genes (Pseudo Genes)	CDSs	tRNAs
*Prevotella sp.* TCVGH	3,061,518	42.6	2569(110)	2409	43
*Streptococcus constellatus* TCV107	1,954,689	38.11	2008(125)	1809	59

**Table 2:** Predicted resistant genes in *Prvotella* sp. TCVGH.

Gene	Coverage (%)	Identity (%)	Detected in Isolated Genome	Detected in Metagenomics
*ErmF*	100	99.38	y	
*tetQ*	99.34	97.91	y	y
*CfxA3*	100	100	y	
*CfxA2*	100	99.9	y	

## Supplementary Materials

The following are available online at https://www.mdpi.com/2077-0383/8/3/351/s1, Figure S1: Taxonomic classification of metagenomic data using partial alignment ratio and read length, Figure S2: Average nucleotide identity analysis of Prevotella sp. TCVGH, Table S1: Sequencing data from Illumina MiSeq, Table S2: MinIon Nanopore sequencing data, Table S3: COG functional CDS of *Prevotella* sp. TCVGH, Table S4: COG functional CDS of *Streptococcus constellatus* TCV107, Table S5: Metagenome analysis of microbial composition, Table S6: Metagenome analysis of fungal composition, Table S7: Metagenome analysis of viral composition, Table S8: Antimicrobial susceptibility testing of *Streptococcus constellatus* TCV107, Table S9: Antimicrobial susceptibility testing of *Prevotella* sp. TCVGH, Table S10: Predicted virulence gene in *Streptococcus constellatus* TCV107, Table S11: Comparison of metagenomic analysis in the study with previous study.
