# Supplementary material for: Complex Microbiome in Brain Abscess Revealed by Whole-Genome Culture-Independent and Culture-Based Sequencing
Source: J Clin Med. 2019 Mar 12;8(3):351. doi: 10.3390/jcm8030351 (PMC6462986; doi:10.3390/jcm8030351)
Supplement: Supplementary file 1 [file jcm-08-00351-s001.zip › Supplementary figures.pdf]

Supplementary Figure S1. Taxonomic classification of metagenomic data using partial alignment ratio and read length.

In order to obtain the taxonomic classification analysis in the metagenomic sequencing, the sequence corresponding results must be identified. The results of sequence align to human genome, the corresponding partial alignment ratio of many sequences is higher than 70%. Sequences less than 500bp will lose recognition because multiple alignment results.

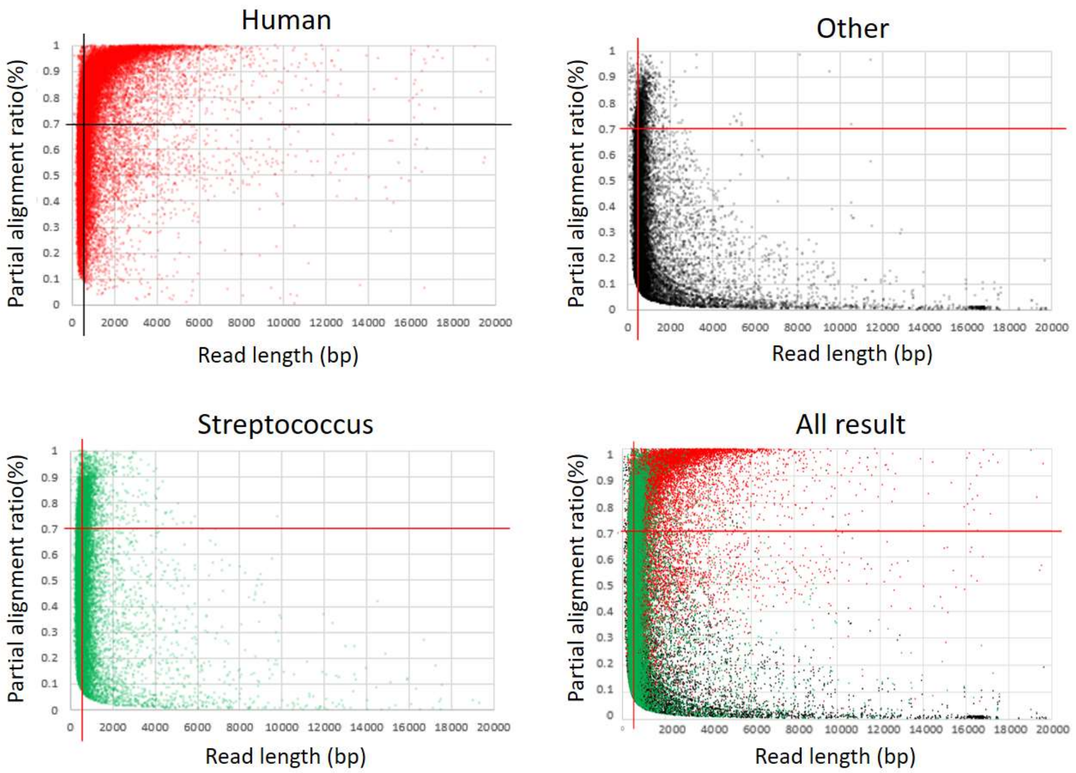

Supplementary Figure S2. Average nucleotide identity analysis of *Prevotella sp.* TCVGH

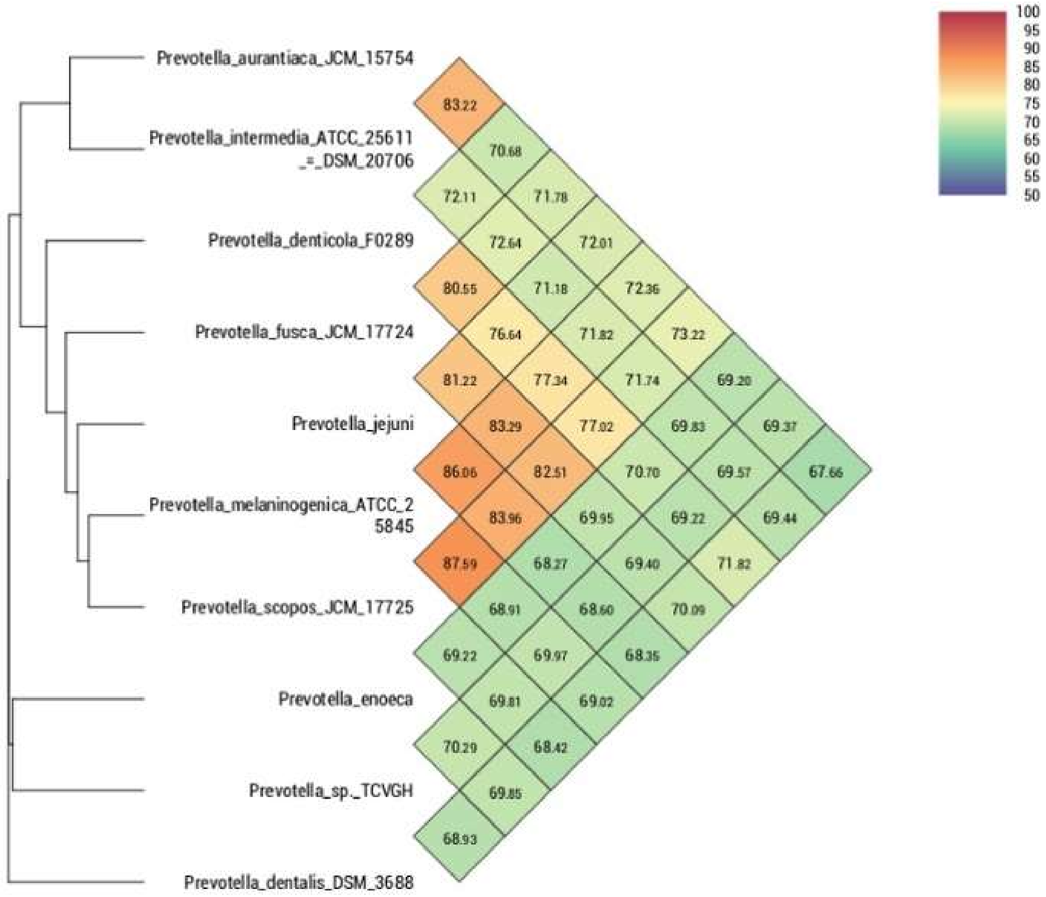

The ANI values of the nine species most closely relate to the *Prevotella sp.* TCVGH. In the MiGA results, the most similar Prevotella enoeca NZ CP013195 also only got 61.61% AAI.

The closest relatives found by MiGA in the database were Prevotella enoeca NZ CP013195 (61.61% AAI) and Prevotella dentalis DSM 3688 NC 019960T (60.0% AAI).

Taxonomic classification

The dataset most likely belongs to the family Prevotellaceae (p-value: 0.0021) and possibly even belongs to the genus Prevotella (p-value: 0.32).

Taxonomic novelty

The dataset most likely belongs to a species not represented in the database (p-value: 0.0046), highest taxonomic rank with p-value ≤ 0.01. It possibly even belongs to a genus not represented in the database (p-value: 0.3), highest taxonomic rank with p-value ≤ 0.5.
