# Supplementary material for: Complex Microbiome in Brain Abscess Revealed by Whole-Genome Culture-Independent and Culture-Based Sequencing
Source: J Clin Med. 2019 Mar 12;8(3):351. doi: 10.3390/jcm8030351 (PMC6462986; doi:10.3390/jcm8030351)
Supplement: Supplementary file 1 [file jcm-08-00351-s001.zip › Supplementary tables.pdf]

Supplementary Table S1. Sequencing data from Illumina MiSeq

Species	read num	avg len	total base	coverage
Prevotella sp. TCVGH	9,778,180	151	1,476,200,000	478x
Streptococcus constellatus TCV107	23,130,094	151	3,491,800,000	1806x

Supplementary Table S2. MinIon Nanopore sequencing data

Sample	read num	n50	total base
Brain abscess (culture-base)	145,810	7,681	6,185,00,000
Brain abscess (culture-free)	286,746	774	158,800,000

Supplementary Table S3. COG functional CDS of *Prevotella sp.* TCVGH

Code	Value	% age	Description
D	24	0.95	Cell cycle control, cell division, chromosome partitioning
M	178	7.07	Cell wall/membrane/envelope biogenesis
N	6	0.24	Cell motility
O	86	3.41	Post-translational modification, protein turnover, and chaperones
T	46	1.83	Signal transduction mechanisms
U	30	1.19	Intracellular trafficking, secretion, and vesicular transport
V	61	2.42	Defense mechanisms
W	0	0.00	Extracellular structures
Y	0	0.00	Nuclear structure
Z	0	0.00	Cytoskeleton
A	0	0.00	RNA processing and modification
B	0	0.00	Chromatin structure and dynamics
J	147	5.84	Translation, ribosomal structure and biogenesis
K	82	3.26	Transcription
L	244	9.69	Replication, recombination and repair
C	73	2.90	Energy production and conversion
E	83	3.29	Amino acid transport and metabolism
F	68	2.70	Nucleotide transport and metabolism
G	108	4.29	Carbohydrate transport and metabolism
H	62	2.46	Coenzyme transport and metabolism
I	39	1.55	Lipid transport and metabolism
P	125	4.96	Inorganic ion transport and metabolism
Q	8	0.32	Secondary metabolites biosynthesis, transport, and catabolism
R	0	0.00	General function prediction only
S	1025	40.69	Function unknown
-	24	0.95	Muti-function
total	2519		

Supplementary Table S4. COG functional CDS of *Streptococcus constellatus* TCV107

Code	Value	% age	Description
D	23	1.19	Cell cycle control, cell division, chromosome partitioning
M	116	6.00	Cell wall/membrane/envelope biogenesis
N	0	0.00	Cell motility
O	53	2.74	Post-translational modification, protein turnover, and chaperones
T	52	2.69	Signal transduction mechanisms
U	26	1.34	Intracellular trafficking, secretion, and vesicular transport
V	59	3.05	Defense mechanisms
W	0	0.00	Extracellular structures
Y	0	0.00	Nuclear structure
Z	0	0.00	Cytoskeleton
A	0	0.00	RNA processing and modification
B	0	0.00	Chromatin structure and dynamics
J	153	7.91	Translation, ribosomal structure and biogenesis
K	145	7.50	Transcription
L	146	7.55	Replication, recombination and repair
C	63	3.26	Energy production and conversion
E	96	4.96	Amino acid transport and metabolism
F	68	3.52	Nucleotide transport and metabolism
G	173	8.95	Carbohydrate transport and metabolism
H	36	1.86	Coenzyme transport and metabolism
I	38	1.96	Lipid transport and metabolism
P	79	4.08	Inorganic ion transport and metabolism
Q	13	0.67	Secondary metabolites biosynthesis, transport, and catabolism
R	0	0.00	General function prediction only
S	567	29.32	Function unknown
-	28	1.45	Muti-function
total	1934		

Supplementary Table S5. Metagenome analysis of microbial composition.

Genus	No. of reads	percent	Genus	No. of reads	percent
Mycobacterium	15,645	18.73%	*Salmonella*	2,186	2.62%
Streptococcus	13,375	16.01%	*Enterobacter*	2,071	2.48%
Pseudomonas	6,014	7.20%	*Staphylococcus*	1,915	2.29%
Bacillus	5,940	7.11%	*Acinetobacter*	1,871	2.24%
Klebsiella	3,639	4.36%	*Listeria*	1,540	1.84%
Escherichia	3,366	4.03%	*Pseudoalteromonas*	1,312	1.57%
Paenibacillus	2,703	3.24%	*Helicobacter*	1,165	1.39%
Shigella	2,534	3.03%	*Alcanivorax*	1,028	1.23%
Bordetella	2,505	3.00%	*Vibrio*	906	1.08%
Chlamydia	2,456	2.94%	*Prevotella*	203	0.24%
Paeniglutamicibacter	2,220	2.66%	Other	8,937	10.70%

Supplementary Table S6. Metagenome analysis of fungal composition.

Genus	No. of reads	percent	Genus	No. of reads	percent
Debaryomyces	596	40.68%	*Aureobasidium*	7	0.48%
Aspergillus	346	23.62%	*Agaricus*	6	0.41%
Malassezia	280	19.11%	*Saccharomyces*	4	0.27%
Mitosporidium	180	12.29%	*Cryptococcus*	2	0.14%
Grosmannia	24	1.64%	Other	20	1.37%

Supplementary Table S7. Metagenome analysis of viral composition.

Genus	No. of reads	percent	Genus	No. of reads	percent
Herpesvirus	30	48.39%	*Retrovirus*	4	6.45%
Moumouvirus	20	32.26%	*Entomopoxvirus*	2	3.23%
Granulovirus	5	8.06%	*Taterapoxvirus*	1	1.61%

Supplementary Table S8. Antimicrobial susceptibility testing of *Streptococcus constellatus* TCV107

Antibiotic	MIC	Interpretation
Penicillin	≤ 0.06	S
Vancomycin	≤ 0.12	S
Erythromycin	≤ 0.12	S
Clindamycin	≤ 0.25	S

Supplementary Table S9. Antimicrobial susceptibility testing of *Prevotella* sp. TCVGH

Antibiotic	MIC	Interpretation
Penicillin	8	R
Cefoxitin	4	S
Clindamycin	8	R
Chloramphenicol	2	S
Metronidazole	4	S

Supplementary Table S10. Predicted virulence gene in *Streptococcus constellatus* TCV107

Gene	Coverage (%)	Identity (%)	Database
hasC	94.32	75.32	VFDB
psaA	89.68	80.36	VFDB

Supplementary Table S11. Comparison of metagenomic analysis in the study with previous study.

Genus	Read number in the study	Case Number in reference	Genus	Read number in the study	Case Number in reference
Streptococcus	13,375	17	*Peptostreptococcus*	8	7
Pseudomonas	6,014	2	*Clostridium*	6	1
Escherichia	3,366	2	*Nocardia*	5	2
Enterobacter	2,071	4	*Treponema*	2	1
Staphylococcus	1,915	3	*Stenotrophomonas*	2	1
Fusobacterium	485	10	*Catonella*	1	1
Enterococcus	211	1	*Tannerella*	1	1
Prevotella	203	4	*Kleibsiella*	0	1
Propionibacterium	194	5	*Tepidimonas*	0	1
Burkholderia	136	1	*Bilophila*	0	1
Bacteroides	94	3	*Citrobacter*	0	1
Eubacterium	92	1	*Dialister*	0	1
Massilia	51	1	*Electronic*	0	1
Porphyromonas	45	4	*Enhydrobacter*	0	1
Filifactor	20	1	*Micrococcus*	0	1
Haemophilus	9	5	*Petrobacter*	0	1
Campylobacter	8	3	*Mycoplasma*	0	1

Al Masalma, M., Lonjon, M., Richet, H., Dufour, H., Roche, P. H., Drancourt, M.,& Fournier, P. E. (2011). Metagenomic analysis of brain abscesses identifies specific bacterial associations. Clinical Infectious Diseases, 54(2), 202-210.
